# Open Dialogue services around the world: a scoping survey exploring organizational characteristics in the implementation of the Open Dialogue approach in mental health services

**DOI:** 10.3389/fpsyg.2023.1241936

**Published:** 2023-11-10

**Authors:** Raffaella Pocobello, Francesca Camilli, Mauricio Alvarez-Monjaras, Tomi Bergström, Sebastian von Peter, Mark Hopfenbeck, Volkmar Aderhold, Stephen Pilling, Jaakko Seikkula, Tarek Josef el Sehity

**Affiliations:** ^1^Institute of Cognitive Sciences and Technologies, CNR, Rome, Italy; ^2^Department of Clinical, Educational and Health Psychology, University College London, London, United Kingdom; ^3^Department of Psychology, University of Jyväskylä, Jyväskylä, Finland; ^4^Department of Psychiatry, Länsi-Pohja Hospital District, Kemi, Finland; ^5^Brandenburg Medical School Theodor Fontane, Brandenburg an der Havel, Germany; ^6^Department of Health Sciences, Norwegian University of Science and Technology (NTNU), Trondheim, Norway; ^7^Independent Researcher, Hamburg, Germany; ^8^Department of Psychology, University of Agder: Kristiansand, Kristiansand, Norway; ^9^Faculty of Psychology, Sigmund Freud Private University, Vienna, Austria

**Keywords:** Open Dialogue, mental health services, self-assessment, peer support, scoping survey, implementation, mental health training, global survey

## Abstract

**Objective:**

This cross-sectional study investigates the characteristics and practices of mental health care services implementing Open Dialogue (OD) globally.

**Methods:**

A structured questionnaire including a self-assessment scale to measure teams’ adherence to Open Dialogue principles was developed. Data were collected from OD teams in various countries. Confirmatory Composite Analysis was employed to assess the validity and reliability of the OD self-assessment measurement. Partial Least Square multiple regression analysis was used to explore characteristics and practices which represent facilitating and hindering factors in OD implementation.

**Results:**

The survey revealed steady growth in the number of OD services worldwide, with 142 teams across 24 countries by 2022, primarily located in Europe. Referrals predominantly came from general practitioners, hospitals, and self-referrals. A wide range of diagnostic profiles was treated with OD, with psychotic disorders being the most common. OD teams comprised professionals from diverse backgrounds with varying levels of OD training. Factors positively associated with OD self-assessment included a high percentage of staff with OD training, periodic supervisions, research capacity, multi-professional teams, self-referrals, outpatient services, younger client groups, and the involvement of experts by experience in periodic supervision.

**Conclusion:**

The findings provide valuable insights into the characteristics and practices of OD teams globally, highlighting the need for increased training opportunities, supervision, and research engagement. Future research should follow the development of OD implementation over time, complement self-assessment with rigorous observations and external evaluations, focus on involving different stakeholders in the OD-self-assessment and investigate the long-term outcomes of OD in different contexts.

## 1. Introduction

Finding its roots in Need-Adapted Treatment (Alanen et al., 1991; Alanen, 1997), OD emerged as an innovative approach within the Finnish Western Lapland mental health services during the 1980s and 1990s. Seven principles became evident during the first research programs and psychotherapy training: (1) immediate help, (2) a social network perspective, (3) flexibility and mobility, (4) responsibility, (5) psychological continuity, (6) tolerance of uncertainty, and (7) dialogism (Seikkula et al., 2001). The first five principles regard the organizational logistics in which mental health services are provided, while the last two refer to the dialogic practice in which mental health professionals engage during network meetings with clients (Seikkula et al., 2003).

Since the 1990s, positive outcomes associated with OD have been documented in Western Lapland (Seikkula et al., 2006). Researchers observed that 82% of patients experiencing acute psychosis following the OD treatment showed no symptoms at the 5-years follow-up. Moreover, 86% of the patients had returned to a full-time job or studies, whereas only 14% were on disability allowance. Encouraging results were also observed during the following decade. A follow-up study confirmed that more than 80% of patients treated with the OD approach were fully employed or engaged in their studies after 2 years (Seikkula et al., 2011). Moreover, the study highlighted a cultural change in the use of the mental health service that led to earlier initiation of treatment, with a shorter duration of untreated psychosis and patients’ first contact happening at a lower age. Findings from a nineteen–year outcomes study indicated that many positive outcomes documented in previous studies are sustained over a long period (Bergström et al., 2018, 2022).

By 2011, OD was “well-established” in Western Lapland but still “little-known elsewhere” (Thomas, 2011). However, in the following decade, the approach started to be applied globally in different contexts and with disparate results. A review which focused on OD implementation in Scandinavia outside of Finland highlighted a significant variety of OD applications that, according to the authors, could be related to the intentional lack of operationalization of the OD principles (Buus et al., 2017). Other authors suggested that the different integrations of the OD approach into clinical practice may depend on the double challenge of introducing a transformation at the individual and the service level (Freeman et al., 2019).

Notwithstanding the heterogeneous panorama of OD applications, the approach has been investigated mainly using a naturalistic research design. The first randomized controlled trial on OD, evaluating the approach’s clinical and cost-effectiveness, was launched in the UK in 2017. The trial is part of the ODDESSI (Open Dialogue: Development and Evaluation of a Social Network Intervention for Severe Mental Illness) research program and compares OD against standard treatment in six mental health services in the UK. Results are expected in 2024 (Pilling et al., 2022).

Overall, the gradual implementation of OD into mental health services has not been described in detail, not even in Finland, despite the breadth of studies reporting on the origin of the approach (Buus et al., 2021). Research focusing on the implementation obstacles has been very scarce for many years, with one study describing organizational challenges observed among the nursing staff in Finland (Haarakangas et al., 2007) and a case study reporting the difficulties of an outreach team practising OD in Denmark (Søndergaard, 2009). More recent research (Gordon et al., 2016; Heumann et al., 2023; Skourteli et al., 2023) highlighted organizational and ideological barriers such as lack of time and resources, rigid professional hierarchy and the burden of working across two different models at the same time (Dawson et al., 2021; von Peter et al., 2023). Although these qualitative studies suggest some adaptation strategies, more global and quantitative research on the implementation of the OD approach is still needed.

Moreover, the fact that the OD approach has not gone through the process of manualisation – that is, the development of a procedure that can be replicated with sufficient uniformity (Waters et al., 2021) poses additional challenges, especially in assessing OD-fidelity. A measure called COMFIDE (Alvarez Monjaras, 2019; Alvarez-Monjaras et al., 2023) was developed as part of the ODDESSI trial to evaluate a good standard of care for community mental health services providing OD and standard crisis and community care. Although more research on OD-fidelity is needed to identify specific and measurable elements (Waters et al., 2021), items and topics from the COMFIDE scale may currently be used for fidelity assessments at a global level.

Different approaches to implement Peer supported Open Dialogue (POD), connecting social and professional networks, have also been described in the last years (Razzaque and Stockmann, 2016; Kemp et al., 2020; Lorenz-Artz et al., 2023). Bellingham et al. (2018) reported that several models of POD had been embedded into clinical practice. In some cases, peer supporters may have a role very similar to that of professional therapists, whereas, in others, they have more limited space. For example, persons with lived experience may not participate in network meetings but be involved as supporters of the community. In other models, they may participate in the network meetings but not attend the reflection spaces addressed only to the clinicians (Bellingham et al., 2018). Due to the heterogeneity of models and scarcity of research on peer workers, a more comprehensive investigation is needed in this area (Kemp et al., 2020).

Pivotal elements in the development of OD services are training, supervision and intervision which need to be “carefully planned” and considered an integral part of the approach (Buus et al., 2017) – intervision is hereby a form of colleague-based supervision practised in Peer-Supported Open Dialogue (see Razzaque, 2019). In Western Lapland, the training of the staff members was one of the three central components of the community psychiatric system (Alakare and Seikkula, 2021), together with the “Family and Team-centeredness” and the research project (Seikkula et al., 2011). Training activities cover theory, supervision, and seminars in which participants are required to analyze their background and family of origin. Experiences of training from different countries, including Norway, the US, the UK, Australia and Italy, have been reported in the literature (Hopfenbeck, 2015; Aderhold and Borst, 2016; Buus et al., 2017; Cubellis, 2020; Florence et al., 2020; Hopper et al., 2020; Jacobsen et al., 2021; Schubert et al., 2021; Pocobello, 2021b). Intervision, intended as a form of colleague-based supervision, and training, including “intentional peer support,” are also part of the activities for peer workers (Hopfenbeck, 2015; Razzaque and Stockmann, 2016; Razzaque, 2019; Hopper et al., 2020). As far as we know, there has been no global investigation on the extent of training and supervision practices in OD services worldwide. Quantitative data on how many people involved in OD services have completed or are completing the training are unavailable. Moreover, the frequency and type of supervision have not been explored so far.

Overall, the requirements for and barriers to the implementation of OD on both the level of organizational structures and staff competencies need to be addressed in research and require a deeper investigation (Mosse et al., 2023).

The present scoping survey was designed to map and explore the existing evidence about the implementation of OD-services globally (Pocobello, 2021a) and to investigate the impact of factors such as OD-training, supervision, research, the involvement of experts by experience and organizational characteristics on services’ OD-self-assessment (OD-SA). In this context, the term “expert by experience” refers to an individual who has/had personal, lived experience with mental health challenges or the mental health care system. This term acknowledges that individuals who have gone through these experiences possess a unique and valuable perspective that can contribute significantly to the improvement of mental health services, policies, and practices (Gupta et al., 2023).

The objectives of the global scoping survey can be summarized as follows:

- To describe services practising Open Dialogue around the globe;
- To pilot testing and validating an Open Dialogue Service Survey Scale including an OD- self-assessment (OD-SA) scale;
- To construct an exploratory model of the organizational predictors of OD self-assessment;
- To provide a measure of teams’ degree of self-assessed adherence to the seven OD principles and
- To identify services ready for outcome evaluation studies.

The study is part of the project HOPEnDialogue,^1^ financed by the Open Excellence Foundation, which aims at investigating the implementation and effectiveness of Open Dialogue in different mental health care contexts around the world.

## 2. Methods

The study is reported according to the CROSS Checklist for Reporting Survey Studies (Sharma et al., 2021) to ensure rigor and credibility.

### 2.1. Study design

We used a cross-sectional study design to collect data from multiple teams providing OD services in mental health care across different countries. The study design involved (1) the development and validation of a OD-self-assessment scale and (2) a quantitatively structured questionnaires to gather information on various aspects of OD services, including their structural characteristics, personnel OD-trainings, as well as practices regarding supervisions, involvement of experts by experience, and research activities.

### 2.2. Ethical clearance

All respondents to the survey have completed an informed consent form embedded in the first page of the questionnaire. A skip-logic survey method was in place in the online form to ensure no collecting of information from respondents who disagreed with the informed consent question. Respondents were informed about the possibility of withdrawing from the survey at any time. Respondents could leave questions not answered.

The survey was not anonymous, since the address of the service and personal contact information of the professional completing the survey on behalf of the OD team was used to check for accuracy and prevent multiple participation. Confidentiality was guaranteed by limiting access to this information to the research team of the ISTC-CNR and saving electronic data on password-protected computers.

Ethical clearance with authorization value was not necessary for this study.

### 2.3. Respondents

Team members of OD-services with leadership responsibility were invited to complete the survey on behalf of the entire facility or OD-team. Individual OD practitioners were excluded.

As the survey is part of the project HOPEnDialogue, it was advertised and primarily distributed through its website Members of the HOPEnDialogue advisory board helped disseminate the survey in their different countries and networks through social media and mailing lists. We have contacted professionals from countries not represented on the board to ask for their support in spreading the survey at a national or local level. The first round of data was collected online using the Survey Monkey platform from January to September 2020. In total, 136 questionnaires were filled out online. The data were exported into Excel. The second round of data collection happened from January 2021 to February 2022 and involved six teams just concluding their foundation training. The questionnaires were filled and sent as PDFs to RP and FC, who added them to the Excel data set. The reason for this late recruitment was related to our intention to include all the services contacting us to have as much as possible comprehensive view of OD implementation globally. In total, 142 services participated in the survey.

### 2.4. Data diagnostics

Data was checked and controlled for consistency. Where available and possible, missing data were completed by checking back with survey responders via email. Of the 142 questionnaires received during the data collection period, the data of 24 OD-services had to be excluded due to incomplete datasets, mainly from the 6th item (clients’ characteristics served in the center) onwards. Often, the unavailability of informants made it impossible to assist in completing the missing questionnaire sections. We undertook a missing data diagnosis on the data from the remaining 118 centers and did not detect systematic patterns (checking summary statistics for variables, counting the number of missing and non-missing values for each variable, correlations to examine if the missingness in one variable is associated with another variable).

### 2.5. Data analysis strategy

To evaluate the statistical validity and reliability of the measurement model of the OD-self-assessment (OD-SA) scale, non-parametric Confirmatory Composite Analysis (CCA; Dijkstra and Henseler, 2011; Schuberth et al., 2018) was calculated with SmartPLS 4® (Ringle et al., 2022). We followed the procedural steps for CCA outlined by Hair et al. (2020). The reliability of the variables was tested using Cronbach’s Alpha and Composite Reliability (ρ_A_).

Descriptive data of the survey have been checked for consistency in Excel spreadsheets and transferred to SPSS^®^ 27.0 (IBM Corp, 2020) for the descriptive and explorative Cluster analysis.

For the descriptive analysis Continuous variables were described using means (M) and standard deviation (SD); for discrete count variables, proportions were reported. The Shapiro–Wilk test was used to assess the normal distribution of continuous variables. As a non-parametric test for differences in group value of *p*s Kruskal-Wallis’ test was used. Association between structural aspects of OD-services was assessed using Loglinear modeling when it concerned the frequency of categorical data (see structural characteristics). The significance level was determined as *p* < 0.05 for all analyses.

For the explorative data analysis bivariate non-parametric correlations were computed between the services OD-SA score and the descriptor variables to identify significant associations.

- To explore structural characteristics of the MHS in which OD-teams emerged and operated, an unsaturated model was chosen using SPSS Statistics’ hierarchical loglinear model selection process with a backwards elimination stepwise procedure;
- To explore professional taxonomies in OD-services hierarchical cluster analysis was used; Provided the sample size of *n* = 118 teams, the number of clusters was estimated to range between *n*/30 = 4 and *n*/60 = 2. To identify equally sized clusters, hierarchical cluster analysis with Ward’s method was used. Count values per variable of the eight professional profiles was standardized to correct for important differences in the counts of personnel in teams. A chi-squared measure of distance was used as a similarity measure;
- A Kruskal-Wallis’ test was calculated to test for significant differences between the OD-teams belonging to different professional clusters. Visual inspection of boxplots was used to assess the similarity of the distributions of OD-SA scores (OD-SA 15) of groups/clusters. Pairwise comparisons were performed using Dunn’s (1961) procedure with a Bonferroni correction for multiple comparisons. Adjusted value of *p*s are presented.

Finally, partial least squares (PLS) regression analysis was conducted to explain the variance of OD-Teams self-assessment scores based on teams- and their services’ characteristics. PLS regression, is a statistical method used in the presence of many predictor variables which may be highly correlated. It is especially useful when the number of predictor variables is larger than the number of observations, a situation where traditional regression methods like ordinary least squares (OLS) struggle (Hair et al., 2018).

The Breusch-Pagan test was used to assess Heteroskedasticity; the PLS algorithm was set to heteroskedasticity consistent standard errors (HC3) to handle the distribution in case of a positive Breusch-Pagan test. HC3 correction calculates robust standard errors that take into consideration the potential heteroskedasticity in the data. It provides more accurate standard errors that are less affected by the presence of heteroskedasticity. This, in turn, ensures that hypothesis tests and confidence intervals derived from the regression analysis are more reliable and valid, even when heteroskedasticity is present (Kaufman, 2013). To deal with missing data the algorithm was set to mean replacement (no weighting vector was used).

### 2.6. Instruments: the Open Dialogue teams survey scale development

RP and TeS developed a first draft of the questionnaire after reviewing the current literature on OD implementation. All authors revised the first draft, and RP further refined the revisions until a consensus was reached. At the end of the development process, 65 questions were finalized for this survey. The full questionnaire is attached as Supplementary material to the article. The items related to OD-team’s transparency, self-disclosure, intervision, intended as a form of colleague-based supervision (Razzaque, 2019) and training were adapted from the OD addendum of the COMFIDE-Questionnaire (Alvarez Monjaras, 2019). The questionnaire was then pilot tested with one OD team, but no changes to the survey content were necessary.

The survey was structured in six sections, each dedicated to an independent dimension of mental health services. In the general part (1) the year the OD-service first started, (2) the presence of other therapeutic models integrated in the mental health service; (3) the age range of patients the OD-service was dedicated to; (4) what diagnostic groups of patients the OD-service works were inquired. Furthermore, three characteristics of the structural domain of mental health services were inquired: (a) the sector to which the MHS belongs [public/other (private, third sector); since the distinction between the private and third sectors was not always clear to respondents, we collapsed these two categories into one category (‘non-public sector’)]; (b) whether the MHS operates as an inpatient or outpatient service, or both; (c) if the MHS is stand-alone- or integrated with other services or other. We further asked about estimating the number of professionals (nurses/occupational therapists/peer-support workers/psychiatrists/psychologists/psychotherapists/social workers /support workers/others) constituting the OD-team.

#### 2.6.1. OD-self-assessment scale: development and validation

For the teams’ OD-self-assessment (OD-SA), we developed 17 items by reviewing the literature on good practice in Open Dialogue. The starting point for the development of the items were the seven principles of OD (Seikkula et al., 2003) with the aim of formulating a minimum of two items for each principle as affirmative statements. Respondents were asked to indicate for each statement the extent to which it reflected the clinical practice in their services over the past 3 months on a five-point Likert scale from 1 = “never,” 2 = “rarely,” 3 = “sometimes,” 4 = “frequently” to 5 = “almost always.” Consequently, higher scores reflected better OD-self-assessment (OD-SA) than lower scores.

#### 2.6.2. Scale validation: confirmatory composite analysis

The content validity of the 17 items composing the OD-self-assessment scale is based on the conceptual review of the OD-Principles formulated by Seikkula et al. (2003).

Discriminant validity refers to the extent to which model constructs may be distinguished from each other. Different to the first five organizational principles, principles 6 (Tolerating Uncertainty) and 7 (Dialogicallity) relate to the way of being and engaging with clients during the network meetings. Due to a low discriminant validity of the two scales – Heterotrait-Monotrait ratio (HTMT) of 0.917 was above the recommended 0.900 threshold (Henseler et al., 2015) – they were merged into one four-item scale of ‘OD-Adherence’ (OD-ADH). For the resulting scales the values of average variance extracted (AVE) exceeded the Fornell and Larcker (1981) criterion (a minimum of 0.5) and HTMT ratio was significantly below 0.90 indicating a good discriminant validity.

Assessing first Cronbach’s alpha reliability of the constructs, it turned out to be ‘good’ for P1 (*r* = 806) and P2 (*r* = 0.806), acceptable for ‘ADH’ (*r* = 0.767) however ‘doubtful’ for P3 (*r* = 0.683), P5 (*r* = 0.632), and ‘not acceptable’ for P4 (*r* = 0.332). Reviewing all factor loadings, we eliminated two critically low loading items (I26: *λ* = 0.52 -> P3; I30: *λ* = 0.63 -> P4) from each of the two scales, turning the P4 scale into a single-item construct consisting of I29 only and the P3 scale into a three-item scale with close to ‘acceptable’ reliability (*r* = 0.698); the internal consistency of P5 (*r* = 0.623) remained low according to the generally applied Cronbach’s Alpha criterion (*r* = 0.705).

New research suggests that the use of a single criterion for established instruments as well as newly explored and developed studies – as the one at hand – may be too conservative for scales developed within the context of the latter (see Hair et al., 2019, p. 9; Hair et al., 2021, p. 119). Composite reliability is therefore recommended for the reliability assessment of newly developed scales (Hair et al., 2018) and the values evidence the scales acceptable level of reliability according to the standards for exploratory studies (see *ρ*_A_ in Table 1).

**Table 1:** Measurement model of the 15 items of the OD-self-assessment (OD-SA) scale: descriptive statistics, factor loadings (**λ**), Cronbach’s alpha, composite reliabilities (**ρ**_A_, and **ρ**_c_), average variance extracted (AVE).

Reliability measures	P1	P2	P3	P4	P5	P6-7: ADH
*M* (*SD*)	**2.96 (1.01)**	**3.80 (0.91)**	**4.51 (0.55)**	**3.83 (1.16)**	**4.29 (0.83)**	**4.10 (0.73)**
Cronbach’s alpha	0.806	0.806	0.698		0.632	0.767
Composite reliability (*ρ_A_*)	0.826	0.808	0.695		0.710	0.774
Composite reliability (ρ_C_)	0.911	0.886	0.832		0.839	0.851
Average variance extracted (*AVE*)	0.836	0.721	0.622		0.724	0.588

Principle	Item	*M*	*SD*	*Λ*	*t*-statistic	*p* value	*VIF*	P1	P2	P3	P4	P5	P6-7: ADH
P1	I18	2.47	0.99	0.897	32.489	0	1.835	0.897	0.493	0.371	0.352	0.120	0.369
P1	I19	3.46	1.21	0.931	62.854	0	1.835	0.931	0.517	0.383	0.337	0.261	0.533
P2	I20	3.36	1.34	0.852	30.298	0	1.815	0.493	0.852	0.377	0.353	0.423	0.424
P2	I21	3.68	1.05	0.854	32.305	0	1.729	0.535	0.854	0.431	0.313	0.317	0.593
P2	I22	4.36	0.79	0.840	21.63	0	1.707	0.376	0.840	0.510	0.338	0.410	0.464
P3	I23	4.64	0.62	0.763	13.651	0	1.299	0.313	0.438	0.763	0.368	0.476	0.455
P3	I24	4.61	0.68	0.825	15.518	0	1.683	0.309	0.341	0.825	0.361	0.330	0.413
P3	I25	4.29	0.77	0.778	12.506	0	1.413	0.348	0.427	0.778	0.236	0.283	0.367
P4	I29	3.83	1.16				1.000	0.375	0.393	0.406	1.000	0.254	0.394
P5	I27	4.31	0.95	0.914	30.719	0	1.271	0.277	0.405	0.431	0.218	0.914	0.501
P5	I28	4.27	0.98	0.782	11.021	0	1.271	0.045	0.362	0.358	0.221	0.782	0.230
P6-7	I31	3.98	1.00	0.790	20.743	0	1.488	0.421	0.472	0.399	0.351	0.448	0.790
P6-7	I32	4.17	0.96	0.753	12.016	0	1.530	0.359	0.392	0.373	0.207	0.261	0.753
P6-7	I33	4.28	0.95	0.803	13.863	0	1.615	0.385	0.490	0.467	0.340	0.362	0.803
P6-7	I34	3.92	0.88	0.718	12.317	0	1.365	0.367	0.428	0.364	0.290	0.304	0.718

*N* = 118.

Shaded values in each column highlight the cross-loadings between items belonging to one scale (=OD-principle).

Multicollinearity appeared not to be an issue for our indicators since each indicator’s Variance Inflation Factor (VIF) value was less than 5. Convergent validity and reliability results are presented in Table 1.

Confirmatory Composite Analysis provided evidence of the measurement model’s construct validity based on the assessment of its convergent and discriminant validity. Nomological validity is confirmed through the positive correlations of the six subscales. The full OD-FID15 scale had a high level of internal consistency, as determined by Cronbach’s alpha of *r* = 0.823. The computed Cramér-von Mises test statistic (*CVM* = 0.16, df = 118, *p* = 0.017) for the composite scores indicated a significant deviation from the normal distribution (skewness = −0.238, kurtosis = −0.203).

## 3. Results

During the timespan January 2020 – February 2022, a total of 142 OD-Teams from 24 different countries responded to the call to participate in the survey. 118 OD-teams (82%) completed the questionnaire responding to the entire OD-self-assessment (OD-SA) scale. We report the number of respondents for each item related to the quantitative data.

### 3.1. Descriptive data

The first OD-mental health services participating in our survey were established in Finland during the 1990ies. This Finnish service remained for about half a decade the pioneering mental health center for the treatment of severe mental illness using OD; in 1995, another center started to offer the OD approach in Norway.

The year 2006 marked a significant turning point in the spread of the OD approach, from where on we observed a stable growth rate of new OD-services of about 24% (SD = 17%) on a yearly basis from five OD-services in Finland and Norway in 2006 to over 100 centers in the year 2020 in 24 countries on five continents (see Supplementary Figure 1).

**Figure 1:**
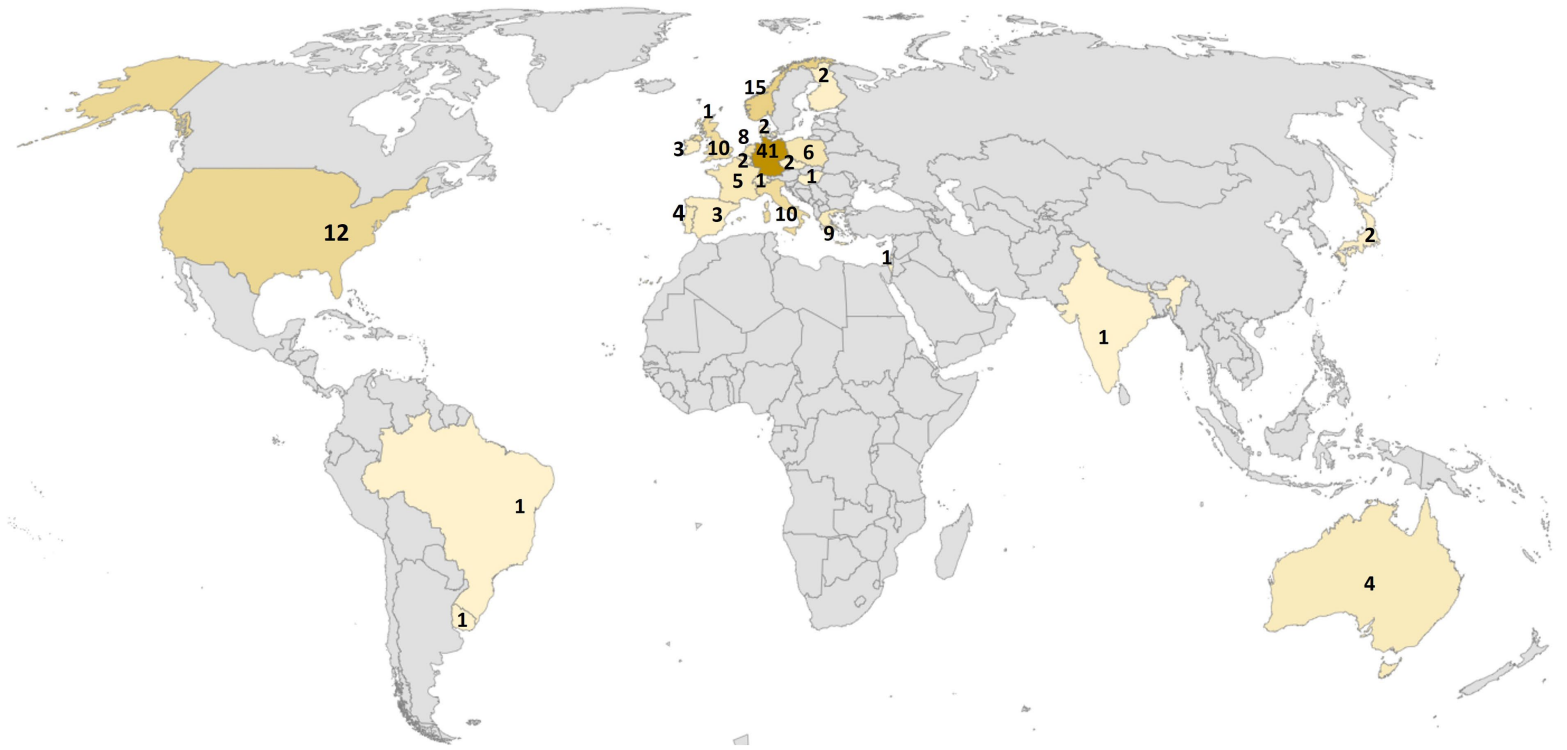
Global map of OD-Teams in mental health services responding to the HOPEnDialogue survey.

Geographically, 85% of OD services were based in Europe, with a presence in almost all North-European countries (except Sweden and Island) and Western Europe (except Austria and Luxemburg; see Figure 1).

#### 3.1.1. Structural characteristics of OD-services

Of the 118 OD-services who completed the survey, 57 (48%) were mental health departments, 42 (36%) were registered associations, 9 (8%) were private practices, and 4 (3%) were foundations; 6 (5%) did not report their legal form of entity. Most teams (62%) belonged to MHS of the public sector, and 45 (38%) OD-teams belonged either to the private sector (*n* = 25) or to the third sector (*n* = 20). None of the teams reported to belong to MHS offering only inpatient service but 42 (36%) offer in-& outpatient service; 76 (64%) offer only outpatient service (see Table 2).

**Table 2:** Observed frequencies and percentages for sector, modality, and type of OD-service.

In which sector is your service?	Modality of services	Integration of services	*n* (%)
Non-public sector [Private- and Third sector (*n* = 45; 21%)]	In- & Outpatient (8)	Integrated service	6 (5%)
		Stand-alone service	2 (2%)
	Outpatient (37)	Integrated service	9 (8%)
		Stand-alone service	28 (24%)
Public sector (*n* = 73; 62%)	In- & Outpatient (34)	Integrated service	29 (25%)
		Stand-alone service	5 (4%)
	Outpatient (39)	Integrated service	27 (23%)
		Stand-alone service	12 (10%)

*N* = 118. Percentages appear in parentheses.

Exploring the structural characteristics associated with the MHS in which OD-teams emerged and operated, resulted in a model including all main effects and two two-way associations: (1) Service Sector * Integration; (2) Service Sector * Service Modality. The likelihood ratio goodness-of-fit test indicated that the model offered a moderate fit to the observed data [*χ*^2^(2) = 4.929, *p* = 0.085]; the specific effects reported in Table 3, however, are mostly significant and support the notion that the structural variables are importantly related (see Table 3).

**Table 3:** Log-linear parameter estimates, values, and goodness-of-fit index for Service Sector, Service Integration, and Service Modality.

Effect	*λ*	*z*	*P*
[non-public sector] X [Standalone]	−1.885	−4.4858	<0.001
[Outpatient] X [Standalone]	1.715	3.622	<0.001
[non-public sector service]	−1.317	−4.531	<0.001
[Outpatient service]	0.028	0.119	0.906
[Standalone service]	−2.389	−5.180	<0.001

G^2^(2, *N* = 118) = 4.929, *p* = 0.085.

We found that the odds of an OD-team belonging to integrated services were 3.73 times higher for OD-teams in public services than for OD-teams in non-public services. Furthermore, a significant association emerged with respect to OD-teams’ Service Integration and Service Modality: the odds for OD-teams working in outpatient MHS not to work in integrated services was 5.71 times higher than for teams working in MHS with in- and outpatient services. The analysis proposes that OD-teams tend to emerge in organizational environments which are public, operate integrated services and offer both inpatient and outpatient services (see Table 4).

**Table 4:** Partial associations for Service Sector, modality of service, and Service Integration.

Effect	Partial association *X^2^* (df = 1)	Sig. (*p*-value)
Service Sector * In-&Outpatient	3.445	0.063
Service Sector * Service Integration (Standalone vs. Integrated)	14.919	<0.001
In-&Outpatient * Service Integration (Standalone vs. Integrated)	8.449	0.004
Service Sector	6.708	0.010
Modality of Service (In-&Outpatient)	9.937	0.002
Integration	4.916	0.027

#### 3.1.2. Access to OD-services and services’ therapeutic context

*Clients-referrals*: Most respondents report referrals to OD-services occur via general practitioners (87%; 90/104); 61% (64/104) of the OD-teams reported referrals from hospitals, and 39% (41/104) referrals from social services. Some services report on established partnerships with associations sharing similar values (e.g., recovery groups) becoming OD-teams’ primary referrals.

An important share of referrals to OD-teams reported are self-referrals: 46% (48/104) report referrals through “word of mouth” or direct requests as described in a comment by a respondent of the Finnish team:

*“Anyone can ask help for anyone (for themselves, for family members, for clients* etc.*) via phone, letters, walking to the office* etc. *Usually, people call the local service number (one number 24/7 for the whole region). Nurses on duty survey what the main problem is and when and where people want to meet (meetings can be arranged within 24 h, but usually, people/patients/clients want the first meeting to be arranged within 2–3 weeks from contact). Then she/he starts to arrange network meetings by calling workers from local outpatient clinics and/or other important people to join the process. Official referrals are not required, but they can be used as well.”*

*Clients-age groups*: Almost all the OD-teams (93%; 110/118) work with clients aged 18–65; about 30% of OD-teams offer their services also to clients under 18 years of age, and about 43% of the OD-services reported an upper age limit of 65 years.

*Clients-diagnostic profile*: Most OD-teams work with clients with psychotic disorders (92%), mood disorders (86%), anxiety and fear-related disorders (81%), to a lesser degree on disorders associated to stress (64%), and other disorders (57%).

*Therapeutic models* mentioned in the OD-services besides the Open Dialogue approach are social psychiatry (10%) and recovery-oriented approaches (9%).

#### 3.1.3. OD workforce

*Hours of teams’ OD practice per week*: An average of 14.2 (Mdn = 10; SD = 12.4) hours per week was reported. 22% (19/87) reported more than 26 h per week of OD practice.

The median number of OD-trained staff members in OD-teams amounted to 14 (S.E. = 2.74) with a median of five members being trained in OD and a median of one member being in OD-training at the time point of study. 61% (72/118) of the teams offered their OD-service less than 20 weekly hours.

Table 5 reports the professional profile of the staff in OD-teams. Using chi-square test of independence, the professional profile of the staff differed significantly between teams operating in the public and non-public sector [*X*^2^(14; 1,604) = 407.793; *p* < 0.001], with clinical personnel such as nurses (34%) and psychiatrists (11%) dominating the OD-teams in the public sector. On the other hand, we found that Support Workers (25%), Social Workers (19%) and Peer-support Workers (13%) dominate the professional profile in OD-teams operating in the non-public sector. Psychologists and occupational therapists contribute equally to both sectors (see Table 5).

**Table 5:** Professional characteristics of the OD-trained workforces.

Items	Categories	*N* = 118	Percent of Cases	Public Sector (*n* = 73)	Non-Public (Private- & Third Sector; *n* = 45)
35. Current number of staff members (Professional profiles of OD-teams); *X*^2^(14; 1,604) = 407.793; *p* < 0.001				*N* = 1,035	*N* = 569
1:	Nurses	439	27%	**353 (34%)**	*86 (15%)*
2:	Occupational Therapists	85	5%	57 (6%)	*28 (5%)*
3:	Peer-support workers	151	9%	*77 (7%)*	**74 (13%)**
4:	Psychiatrists	139	9%	**116 (11%)**	*23 (4%)*
5:	Psychologists/Psychotherapists	263	16%	**188 (18%)**	75 (13%)
6:	Social workers	271	17%	163 (16%)	**108 (19%)**
7:	Support workers	178	11%	*33 (3%)*	**145 (25%)**
8:	Others	78	5%	48 (5%)	30 (5%)
Number of staff in OD-Teams (*n* = 72)		*M* = 19.83; S.E. = 2.74; Median = 14.0;			
Caseload size currently (*n* = 72)		Median = 15.5;			
*Maximum caseload (*n* = 72)		Median = 30.0;			
*Number of Staff-training in progress (*n* = 72)		*M* = 4.19; S.E. = 1.63; Median = 1.0			
Number of OD-trained staff in teams (*n* = 72)		*M* = 8.71; S.E. = 1.37; Median = 5.0			

Categories significantly underrepresented are indicated in italic (adjusted residual < −1.96 at *p* < 0.05); categories significantly overrepresented are indicated in bold (adjusted residual > 1.96 at *p* < 0.05). *Median values of Maximum caseload and Caseload are in many cases based on subjective estimates only since (especially) public services in many countries are required to offer services as requested.

##### 3.1.3.1. OD team taxonomy

To explore potential taxonomies of professional configurations in OD-teams, we ran a cluster analysis based on the standardized counts of professionals in each of the eight professional categories in 118 teams. Ward’s linkage method with chi-squared distance metric was employed for the hierarchical clustering process. Missing values were treated as missing in the analysis.

The agglomeration schedule revealed that clusters were formed in 95 stages, with Ward’s linkage coefficients ranging from 0.000 to 31.171. A dendrogram was utilized to visualize the hierarchical structure of the data clusters (see Supplementary Figure 2) and cluster membership for each case was saved in a new variable. Coefficients increased moderately from 16.987 to 17.659 to 18.463, and then took a much larger leap from 22.888 to 25.236, and then another jump from 27.993 to 31.171 which indicated a good cut-off point at 27.993 with four clusters of OD-teams based on the following professional characteristics (see Table 6):

**Table 6:** OD-team taxonomy: Professional profiles of each cluster.

Professional profiles	Cluster: “Multi-Professionals team” (*n* = 17)	Cluster: “Clinical Psy-team” (*n* = 33)	Cluster: “Nurses and Occupational therapists team” (*n* = 30)	Cluster: “Social worker team” (*n* = 16)	All
Psychologists/psychotherapist	15%	**35%**	18%	11%	22%
Psychiatrists	11%	19%	9%	0%	11%
Nurses	17%	16%	**24%**	**23%**	19%
Social workers	12%	13%	11%	**47%**	18%
Peer-support workers	**11**%	8%	**11%**	9%	9%
Support workers	8%	5%	5%	3%	5%
Other professions	**24%**	0%	0%	2%	5%
Occupational therapists	3%	0%	**13%**	2%	5%
Prof. heterogeneity score (M) (SD)	**5.6**; 2.0	3.7; 1.5	**5.1;** 1.3	3.6; 1.6	4.5; 1.8
OD-FID15 (Mdn)	4.2	4.1	**4.3**	*3.6*	4.2

Categories significantly underrepresented are indicated in italic (adjusted residual < −1.96 at *p* < 0.05); categories significantly overrepresented are indicated in bold (adjusted residual > 1.96 at *p* < 0.05).

- “Multi-professionals teams” (*n* = 17): are characterized by the highly heterogeneous professional profile in which 5–6 professions are on average presented;
- “Clinical Psy-Teams” (*n* = 33): are dominated by clinical professions (psychologists/psychotherapists, psychiatrists, and nurses) with a low degree of professional heterogeneity;
- “Teams with a prevalence of Nurses and Occupational therapists” (*n* = 30): are characterized by the highest share nurses, occupational therapists and peer-support workers;
- “Teams with a prevalence of Social workers” (*n* = 16): are dominated by the highest share of social workers (47%), a high share of nurses (23%), and it is the only group characterized by the absence of psychiatrists (0%).

Peer-support workers were represented equally in all clusters, with a share of about 10%.

Exploring whether the OD-teams with professional profiles differed in their OD-SA score, revealed that median scores were statistically significantly different between the different clusters [*χ^2^*(3) = 13.816, *p* = 0.003]: “Teams with a prevalence of Social worker” (*Mdn* = 3.58) scored statistically significantly lower on the OD-SA scale (OD-FID15) than “Multi-professional teams” (*Mdn* = 4.20; *p* = 0.030) and also lower than “Teams with a prevalence of Nurses and Occupational therapists” (*Mdn* = 4.28; *p* = 0.002) but not with respect to “Clinical Psy-teams” (*Mdn* = 4.07; *p* = 0.146). OD-teams composed of multiple professions yielded significantly higher OD-FID15 scores [*χ^2^*(3) = 20.571, *p* < 0.001; see Table 6].

#### 3.1.4. OD staff training

1,192 staff members were reported to have taken recognized OD-training. Furthermore, 448 OD trainings were undertaken at the time of the survey, so a 38% growth rate of active OD practitioners could be projected for the upcoming years.

With respect of the share of OD-trained personnel in services:

- 4 = 26% (*n* = 27) of the OD-teams had all their clinical staff trained or undergoing a recognized OD-training program;
- 3 = 15% (*n* = 16) had only a small number of exceptions (e.g., a couple of members of staff who have recently joined, but are expecting to start training soon) not being OD-trained;
- 2 = 17% (*n* = 18) had most clinical staff completed or are undergoing a recognized OD training, and most of the remaining staff were due to be trained soon;
- 1 = 42% (*n* = 44) had less than half of the clinical staff with OD-training completed or were undergoing a recognized Open Dialogue training.

The item was scaled on a four point Likert scale ranging from 1 (less than half) to 4 (all their clinical staff trained) resulting in an OD-training level score (*M* = 2.26; *SD* = 1.24).

Responding to the question “Did the training include some self-work on participants’ family of origin?” 45% (*n* = 48) of the teams reported having it included for all the practitioners trained; 13% (*n* = 14) for most; 12% (*n* = 3) only a few; and 11% for none.

Concerning the types of OD-trainings undertaken we report first (a) the percentage relative to the number of trainings and second (b) the percentage of teams who reported at least one member to have taken this training:

(1) 1-year “Open Dialogue practitioner foundation training”
  - 80%; *n* = 911;
  - 67% of the OD-teams.
(2) 3-years “Full Open Dialogue practitioner training”:
  - 12%; *n* = 132;
  - 14% of the OD-teams;
(3) “Peer-supported Open Dialogue social network” (duration: 1 year):
  - 8%; *n* = 91;
  - 11% of the OD-teams;
(4) “Trainers’ training program” (duration: 2 years);
  - 4% (*n* = 50);
  - 23% of the OD-teams;
(5) “International certification training in dialogic practice” (duration: 1 year):
  - 1% (*n* = 8);
  - 7% of the OD-teams.

To assess the OD-training level of teams, the number of training-years was divided by the number of trainings reported per team. On average, each OD-team was endowed with a mean of 1.1 years (S.D. = 0.72) of OD-training.

#### 3.1.5. OD supervision and intervision

66% (*n* = 78) of the OD-teams reported having supervision in place to help clinicians reflect on and develop their OD-practice. 34% (*n* = 40) organize their supervision at least weekly, 25% (*n* = 29) at least monthly and 27% (*n* = 32) report supervision at least once every 3 months.

Supervisions include (1) mainly practitioner reflections (92%; *n* = 65) which (2) are in 73% (*n* = 52) of the teams observed and then reflected by other team members; (3) 58% (*n* = 41) of the teams include final reflections at the end of supervisions (e.g., original pair/group share a final reflection at the end); (4) 35% (*n* = 25) include a brief mindfulness practice during their supervision. We calculated a supervision score ranging from 0 (no supervision) up to five (supervision including all the four listed supervision activities) to measure teams’ OD-supervision practice (*M* = 3.05; SD = 1.76).

Next, to supervision meetings, intervisions in the form of team meetings to reflect on Open Dialogue practice occur at least weekly in 28% of the teams; 33% report at least monthly meetings; 26% meet at least once every 3 months for this purpose.

#### 3.1.6. Research capacity

20% of the OD-teams reported belonging to service including research and development units, and 68% collaborated with universities and external research institutions; 44% have already been involved in research programs.

Most OD-teams collected data about their clients’ sociodemographic (e.g., gender, age), mental health (+95%), psychiatric history (86%), and medication (85%), and only 35% collected data on clinical routine outcomes. However, less than half of the teams used these data to evaluate clients’ and/or carers’ service satisfaction (46%) and service evaluation (47%).

Open Dialogue services reported (1) to be involved in audits (28%), (2) evaluations (32%), (3) quality improvement programs (47%) and (4) research programs (65%). A sum score ranging from 0 (not involved in any research or other systematic service evaluation programs) – 4 (all of the items) was calculated for the variable of teams’ ‘Research Capacity’ (*M* = 0.98; *SD* = 1.13). The low mean value reflects the data of 43% of teams (excl. 13 missing values) not being involved in any research or other systematic service evaluation programs (Score = 0).

#### 3.1.7. Peer- involvement of experts by experience

In 56% (*n* = 60/118) of the OD-teams, experts by experience contributed to the OD-service. About 160 experts by experience were reported in this survey to practice Open Dialogue in these teams where they are primarily involved in the delivery of care (86%), development and planning (70%) and training as trainees (66%) in services. Less often, they are trainers in teams (46%) or engaged in evaluating and assessing services (43%).

48% of OD-teams recognize experts by experience formally in their role as paid workers of the service, while 11% of OD-teams report experts by experience to contribute to their services as volunteers. 44% are involved in supervision like other members of the team and 32% receive psychological support or dedicated supervision.

In 13% of the teams experts by experience participate in all network meetings; 41% engage them in reflections; in 35% join as support for the service user or family network, and in 30% of the teams they are involved as facilitators and moderators in meetings.

### 3.2. Exploring organizational antecedents of OD-self-assessment: partial least square multiple regression analysis

Zero-order correlations were computed to examine the associations between OD-services’ characteristics and their OD-SA score (see Table 7). The following service characteristics were significantly positively correlated with teams’ OD-SA Score: (1) Share of OD-Training in Staff, (2) Supervision for OD Practice, (3) Research & Evaluation, (4) Experts by Experience (EXBEX) involved in Supervision, (6) Teams’ professional heterogeneity, and (7) Clients’ self-referrals to services; negatively correlated were (8) Clients’ average age groups (see Table 7) and remained significant as predictors in the multiple regression model. Fitting the regression model, two more items emerged as significant predictors: (5) the role of EXBEX as Facilitators, (9) Service Modality: Outpatient (see Table 8).

**Table 7:** Means, standard deviations, and intercorrelations for OD-self-assessment (OD-SA) measure and OD-team characteristics predictor variables.

	*M*	SD	1	2	3	4	5	6	7	8	9
OD- self-assessment (OD-SA) Score	3.98	0.60	0.31**	0.38**	0.34**	0.24**	−0.15	0.26**	0.21*	−0.30**	0.11
1. Share of OD-Training in Staff (I50)	2.26	1.17	---	0.20*	0.17	0.05	0.08	−0.17	0.03	−0.09	0.08
2. Supervision for OD Practice (I51-2)	2.51	1.76		---	0.45**	0.12	0.1	0.25*	0.06	−0.17	−0.08
3. Research & Evaluation (I41)	0.98	1.06			---	0.13	0.12	0.35**	0.04	−0.04	−0.11
4. EXBEX involved in Supervision (I63)	0.79	0.31				---	0.25*	0.05	−0.01	0.17	−0.02
5. EXBEX: Facilitator (I62.1)	0.53	0.37					---	0.01	−0.03	0.07	0.01
6. Service: Team heterogeneity (I18)	4.45	1.58						---	0.01	−0.11	−0.21*
7. Service: Self Referrals (I12)	0.46	0.47							---	−0.11	0.06
8. Service: Clients’ Characteristics: Average Age (I6)	40.64	6.93								---	0.05
9. Service: Outpatient	0.36	0.48									---

*p < 0.05; **p < 0.001.

**Table 8:** PLS multiple regression analysis summary for variables predicting OD-Teams’ OD-self-assessment (OD-SA): *F*_(9, 108)_ = 10.727, *p* < 0.001.

OD-Self-Assessment	*B*	*SE B*	*B*	*SE*	*t*	*p*	*VIF*
1. Share of OD-Training in staff (I50)	0.13	0.04	0.25	0.04	3.31	0.001	1.15
2. OD-Supervision and Intervision (I51-2)	0.06	0.03	0.17	0.03	1.94	0.055	1.35
3. Research capacity (I41)	0.08	0.04	0.15	0.04	2.17	0.032	1.43
4. Peer involvement in supervision (I63)	0.57	0.13	0.29	0.13	4.26	0.001	1.12
5. Peers-role as Facilitator (I62.1)	−0.42	0.11	−0.26	0.11	3.78	0.001	1.09
6. Service: Teams’ professional heterogeneity (I18)	0.08	0.03	0.20	0.03	2.35	0.020	1.29
7. Service: Self Referrals to services (I12)	0.18	0.09	0.14	0.09	2.01	0.047	1.02
8. Service: Clients’ Characteristics: Average Age (I06)	−0.02	0.01	−0.24	0.01	3.59	0.001	1.10
9. Service: Outpatient service (I04)	0.21	0.09	0.17	0.09	2.37	0.020	1.06

*R*^2^ = 0.47. *N* = 118. *p* < 0.001.

Exploratory partial least squares (PLS) regression analysis was used to identify significant predictors explaining the variance of teams OD-SA scores. The overall PLS-model for teams’ OD-SA (operationalized via the 15 items score) was found to be statistically significant, *R^2^* = 0.421, (*R^2^_adj_* = 0.384; *p* < 0.001), accounting for 42% of the OD-SA measurement variance with a statistically significant model [*F*_(9, 108)_ = 10.727, *p* < 0.001].

- “*Share of OD-Training in Staff* “(see Section 3.1.4) was found to have a significant positive relationship with Teams’ OD-SA, *β* = 0.25, *t*(108) = 3.31, *p* < 0.001. For every one-unit increase, the OD-SA score increased by 0.24 units, controlling for the effects of the other independent variables.
- “*Supervision for OD-practice*” (see Section 3.1.5) showed a statistically only moderate relationship with teams’ OD-SA [*β* = 0.17, *t*(108) = 1.94, *p* = 0.055], holding all other independent variables constant.
- “*Research capacity*” (see Section 3.1.6) demonstrated a significant positive relationship with OD-SA [*β* = 0.15, *t*(108) = 2.17, *p* = 0.032].
- “Peer-involvement in OD-practice” (see Section 3.1.7) was not correlated with teams’ OD-SA. However, one single item “*EXBEX involvement in supervision*” (4) was positively correlated [*β* = 0.29, *t*(108) = 4.26, *p* < 0.001] and one other “*EXBEX role as facilitator*” (5) was negatively correlated [*β* = −0.26, *t*(108) = 3.78, *p* < 0.001] with OD-SA.
- (6–9) four service characteristics emerged as significant predictors of teams’ OD-SA: The *presence of multiple professions* in an OD-team (6) appeared to be positively correlated with OD-SA: *β* = 0.20, *t*(108) = 2.35, *p* = 0.020, so that the presence of one more different professions in OD-teams increases the OD-score by 0.20 points all other independent variables kept constant. Furthermore, the possibility of *Self-referrals* to OD-services is likely to increase its OD-SA by 0.14 [*β* = 0.14, *t*(108) = 2.01, *p* = 0.047]. Also, it appears that *clients’ age-groups* to which OD-services are dedicated are negatively correlated to OD-SA [*β* = −0.24, *t*(108) = 3.59, *p* < 0.001], meaning that OD-services working with younger clients tend to operate more according to the seven OD-principles than OD-services working for older clients. Finally, OD-services operating as *outpatient services* appear to be slightly facilitated in their work according to the OD-principles [*β* = 0.17, *t*(108) = 2.37, *p* = 0.020].

**Figure 2:**
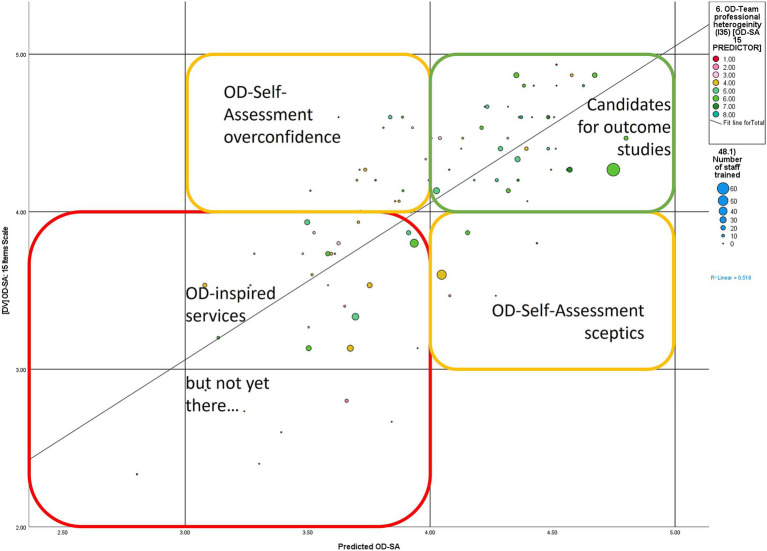
Scatterplot of predictors of OD-self-assessment and teams’ OD-self-assessment.

The predictor scores of OD- self-assessment were projected into a scatterplot to identify OD-teams which may represent potential candidates for a mental health outcome study of Open Dialogue treatment (see Figure 2).

The scatterplot in Figure 2 offers a topological synopsis of OD-teams in four quadrants to capture the plausibility of the OD-SA scores.

1. Cases of “OD- self-assessment overconfidence” (see yellow quadrant above the regression in Figure 2): high self-assessment of OD-SA when conditions appear to be unfavorable. Here recommendations to work on OD-pillars such as training, supervision, research and/ or peer involvement may apply before outcome studies may be considered.
2. Cases of “OD-self-assessment sceptics” (see yellow quadrant below the regression in Figure 2): If all OD-pillars are in place, why is there a low OD- self-assessment? Further investigation is needed to better understand these cases.
3. “OD-inspired services”: These are cases along the lower end of the regression (see red quadrant in Figure 2), which do have issues with the antecedent conditions to offer the OD approach (OD training, supervision, etc.) and self-assess their OD practices low.
4. “Candidates for outcome studies”: these cases along the upper end of the regression (see green quadrant in Figure 2) appear to dispose of the favorable condition to provide OD practice and self-assess their OD-practice high. Considering the self-assessment nature on which these data are based, further assessment by independent evaluators might be needed to understand their current state of organizational and clinical practice prior to commencing an outcome study.

## 4. Discussion

The first achievement of this study was to develop, pilot and validate a scale for the self-assessment of mental health care services regarding the seven Open Dialogue principles. Our results demonstrated the construct validity of the measurement model, confirming the reliability of its subscales (OD principles) and their convergent and discriminant validity. However, further development is needed to improve the subscales related to Responsibility and Psychological continuity.

The empirical results of our global survey provide valuable insights into the characteristics and practices of OD teams across different countries. The results indicate a stable growth in OD-services over time (as indicated via the dates when they were first established), with a steady increase from five services in Finland and Norway in 2006 to over 100 centers in 2020 across 24 different countries. Geographically, the majority of OD-service centers were based in Europe, particularly in North European and Western European countries. This suggests that OD has gained significant traction in these regions, potentially due to cultural factors, research support, or policy initiatives promoting its implementation (e.g., Gooding, 2021; WHO TEAM – Mental Health and Substance Use, 2021).

The structural characteristics of OD services varied, with mental health departments and registered associations being the most common types of entities. Most OD-teams belonged to the public sector, while a significant portion belonged to the non-public sector. OD-teams operating in outpatient mental health services were more likely to work in integrated services compared to teams in inpatient services. This diversity in organizational settings highlights the adaptability and flexibility of OD within different healthcare contexts, which can help expand access to OD for a broader population.

Referrals to OD-services primarily came from general practitioners, hospitals, and social services, potentially indicating that OD is perceived as a valuable option by various stakeholders involved in mental health care. Self-referrals, through word of mouth or direct requests, also played a significant role. Furthermore, self-referrals reported by 46% of the teams were a positive predictor of OD-SA scores. The positive correlation between self-referrals and OD-SA scores suggests that individuals who actively seek out OD services may benefit from the approach, emphasizing the importance of client-centered care and empowerment. Furthermore, self-referrals may indicate the impact of OD-teams ‘reputation’ so that they are recommended by former clients or other professionals.

The survey findings confirm further that OD is utilized for a wide range of diagnostic profiles, with a particular emphasis (92%) on treating psychotic disorders. This aligns with previous research highlighting the positive outcomes of OD in this domain (Seikkula et al., 2003, 2011; Putman and Martindale, 2021). However, the survey findings also indicate the treatment of various other diagnostic profiles, indicating the versatility and applicability of OD beyond psychosis, which expands its potential impact on mental health care.

OD-teams worked with clients across different age groups ranging from minors (< 18) up to the elderly (+65). This suggests that OD is not limited to specific age ranges or diagnoses, indicating its versatility in addressing a wide range of mental health concerns. However, the significant negative correlation of OD-SA with client’s age groups also indicates that teams working with younger clients tend to operate more in line with OD principles which might be associated with the fact that programs which address first-episode psychosis typically serve adolescents and young adults (Gidugu et al., 2021). Another explanation may be that older clients have usually been in the help system for a long time. This means that the private social network has usually already withdrawn and is more difficult to activate – bringing about low scores on the OD-SA scale according to the second principle of networking. Furthermore, the longer patients have been cared for, the more they might have become accustomed to professional care and the less socially inclusive ideas and steps come from the network itself. The latter may then lead to more action orientation of the team or through other care structures around the client.

Apart from a few services entirely organized with OD-trained professionals, the average number of OD-trained professionals involved in services is around 11.5, thus, representing primarily small OD teams. Moreover, only 22% of teams reported practising OD more than 26 h per week, which confirms that most professionals are practising OD alongside other approaches, with a risk of additional burden (Dawson et al., 2021). Depending on how different the treatment philosophy of the other part of the work is compared to OD, this can result in a real obstacle in the ability to maintain and keep a dialogical attitude. Furthermore, it needs to be better understood how some professionals can define specific times for “practicing OD” and times when they do not. This is in contrast with the Model in Western Lapland which is more alike a treatment culture and a way of arranging the entire service to guarantee dialogical responses to people’s difficulties, rather than a specific method (Seikkula, 2013). Maybe this finding evidence challenges in defining what OD is, as well as mental health professionals’ tendency to operationalize or view it as a treatment method/technique, when it may simultaneously lose some essential “healing” elements of care (von Peter et al., 2023).

The OD workforce consisted of professionals from various backgrounds, including clinical personnel (psychiatrists, psychologists and psychotherapists and nurses), support workers, social workers, and peer-support workers. The professional profile of the staff differed between OD-teams operating in the public and non-public sectors. Cluster analysis identified four distinct clusters of OD-teams based on professional characteristics, showing that OD-teams composed of multiple professions had higher OD-SA scores compared to teams with a more specific professional composition. This finding is consistent with previous research emphasizing the value of interdisciplinary teamwork and the need for integrated care approaches (Montesano and Scherb, 2023). For instance, multidisciplinary mental health service models have demonstrated a positive impact in improving client engagement and communication among different specialties (Killaspy et al., 2009). Moreover, providers have indicated that various skills and perspectives contributed to increasing the range of solutions, with final benefits for the service users (Odden et al., 2019).

In terms of training, previous research has identified training costs and length as a barrier to implementation (Gordon et al., 2016; Florence et al., 2020), but there were no data available about the number and share of professionals in the teams participating in accredited training, which varies considerably according to the survey results. On the one hand, teams are practising OD with all (26%) or almost all (15%) of the staff have received accredited training and teams with most of the professionals trained (17%); on the other hand, there are as many as 42% of teams practising OD with less than half of the professionals trained. This clearly differs from Western Lapland, where most OD professionals have a three-year dialogical training (Alakare and Seikkula, 2021; Putman, 2021) whereas the most common training program reported in our survey was the 1-year “Open Dialogue practitioner foundation training.” We also observed an indication of substantial growth since about 38% of staff members were still in training at the time point of the survey.

The survey at hand suggests that supervision is an important component of OD implementation as indicated by its near significant role as a predictor of teams’ OD-SA scoring (see Table 8). Supervision activities reported in the survey included practitioner reflections, observations and reflections by other team members, final reflections, and mindfulness practice. Regarding frequency, 66% of OD-teams reported having periodic supervision in place to support clinicians in reflecting on and developing their OD-practice. However, 22% indicated no supervision, and 10% did not respond to this question. This lack of supervision can be particularly critical, especially considering the documented limitation in training level and percentage of professionals trained in the different teams. Challenges with OD supervision were already reported in previous studies (Hopper et al., 2020). Intervisions in the form of team meetings to reflect on OD-practice took place regularly had however no statistically significant impact on teams’ OD-SA scoring.

Research and evaluation are an integrative part of the development of OD in Western Lapland (Seikkula et al., 2011), as confirmed in this survey, where their team represented an outlier on this topic. The mean score of international teams remains relatively low (0.98 on a scale from 0 to 4), since 38% of teams are not involved in any research or evaluation programs, and only a minority belonged to services with research and development units. Data collection focused on sociodemographic information, mental health and psychiatric history, medication, and to a lesser extent, routine clinical outcomes.

The survey findings indicate that experts by experience are involved in approximately 52% of the OD-teams surveyed. However, the extent of their involvement varied across teams. These experts are primarily engaged in the delivery of care, development and planning, and training within the services. Still, consistent with previous research (Bellingham et al., 2018), their systematic involvement in network meetings is limited, with only 21% of teams with experts by experience reporting their participation. The results also reveal that around 48% of OD-teams formally recognize experts by experience in their role as paid workers, while 11% rely on them as volunteers. Furthermore, we found contradictory results related to the impact of peers’ involvement on OD-SA scoring. On the one hand, OD-teams in which experts by experience were involved in supervision were positively correlated with high scores on the OD-SA scale, underscoring the potential benefits of their inclusion in team dynamics. On the other hand, we found a negative correlation between the peers in the role of facilitators of network meetings and OD-SA scores, which would need further investigation and may be related to the difficulties of peers in accessing training compared to mental health professionals. Other possible interpretations could be the lack of role clarity that represents a barrier to establishing peer support (Crane et al., 2016), clinical hierarchies in mental health services (Razzaque and Stockmann, 2016), or the difficulty for peers to align with treatment routines that have been developed in a professional context (von Peter et al., 2021).

Finally, a major achievement of the study was to identify several organizational characteristics that significantly correlate with OD fidelity, including staff OD-training share, supervision for OD practice, research capacity, professional heterogeneity, self-referrals, outpatient services, and the involvement of experts by experience. These findings highlight the importance of these factors in promoting fidelity to the OD approach and suggest strategic areas for intervention and improvement to support OD implementation globally.

### 4.1. Limitations and recommendations for future research

The first limitation of the global survey is related to the sample’s representativeness. In fact, despite our efforts to advertise the survey internationally, its reach may have been limited, potentially excluding certain regions or countries where OD is practised, such as Sweden and other teams in Norway. As a result, the findings may not fully represent the global landscape of OD teams.

The second limitation is related to the accuracy and the representativeness of the obtained results since only one member from each OD service has filled out the survey, and his/her view may have been different compared to other team members. Therefore, we recommend that future research include more perspectives and evaluations, inviting different stakeholders to assess the same service, similar to what Price et al. (2020) did in a different context. Moreover, as the survey relied on self-reported data, respondents may have been less accurate and positively biased (Martino et al., 2009) and provided socially desirable responses, either unintentionally or deliberately.

The third limitation is related to the fact that the survey employed a self-assessment scale developed specifically for the study. While efforts were made to ensure the statistical validity and reliability of the self-assessment scale, the items may not fully capture all dimensions of OD fidelity, or there may be conceptual limitations in how fidelity is measured and assessed. This could affect the accuracy of the self-assessment scores reported by the teams. Therefore, the questionnaire used can only be considered preliminary work for an OD fidelity scale validation study to be conducted according to standardized measurement methodology (i.e., Bond and Drake, 2020).

Finally, the survey is cross-sectional and based on quantitative data. This also implies that important information from OD services that are not active anymore are missing. We recommend future longitudinal studies to provide insights into the development of OD services over time and the use of qualitative investigations to gain a deeper understanding of the experiences and perspectives of OD teams, service users, and experts by experience and capture contextual information about the challenges and facilitators of implementing OD, including aspects that have not been assessed in the survey such as financial resources and team dynamics.

## 5. Conclusion

The survey findings contribute significantly to advancing the knowledge and understanding of the global development of Open Dialogue in mental health services. Also, indicating a growing number of OD services across different countries, the survey results demonstrate an increasing recognition of the value of OD in mental health care but also the urgent need for concrete actions to ensure its appropriate implementation.

Specifically, the global scoping survey can inform mental health policymakers and organizations to consider the following critical areas of intervention:

- Training: The survey highlights variations in OD training among professionals within OD teams, suggesting that mental health organizations and educational institutions should collaborate to develop and provide accredited OD training programs that cover various professional backgrounds and ensure a high level of competency among professionals delivering OD.
- Supervision: the survey reveals that many OD teams do not have regular supervision. As supervision plays a role in maintaining and improving fidelity, especially for teams at the beginning of their practice, mental health organizations and policymakers should provide support and resources for teams to engage in regular supervision.
- Research: the survey reveals that research and evaluation activities in OD are relatively limited globally. Encouraging and supporting research and evaluation in OD can contribute to the evidence base and help investigate OD interventions’ effectiveness, cost-effectiveness, and outcomes. Mental health organizations, funding agencies, and researchers should prioritize research on OD, promote collaboration among international research teams, and allocate resources for rigorous evaluation studies to build a stronger evidence base, not only on psychosis.
- Involvement of experts by experience: the survey findings suggest that involving peers in OD supervision positively correlates with OD-SA scale, highlighting the importance of meaningful involvement and engagement of service users in delivering mental health services. However, the findings also indicate potential difficulties for peers to facilitate network meetings in adherence to the OD-principles. Mental health organizations should actively support the participation of experts by experience in training and supervision to overcome this difficulty in their involvement.
- Mental Health Settings: the survey findings indicate that OD is primarily practised in outpatient settings and focuses on the treatment of psychosis. Mental health organizations should explore opportunities to integrate OD principles and practices into other mental health care settings, such as inpatient units, community clinics, and primary care settings. This expansion would allow a broader range of individuals with mental health needs to benefit from OD’s person-centered and dialogical approach.

Finally, the survey highlights the geographic concentration of OD services in certain regions, particularly in Europe. There is a need to promote collaboration and knowledge exchange among OD teams globally to share best practices, experiences, and research findings.

## Data availability statement

The datasets presented in this article are not readily available because data would identify the participating entities. Requests to access the datasets should be directed to Raffaella.Pocobello@istc.cnr.it.

## Author contributions

RP was involved in all the research process. TeS contributed to developing the questionnaire, data analysis, writing, and interpretating results. FC contributed to the literature review, data entry, writing, and interpreting results. MA-M, TB, SeP, MH, VA, StP, and JS contributed to the development of the questionnaire, the interpretations of the results, and reviewing the final version of the manuscript. All authors approved the submitted version of the article.

## Footnotes

^1^https://www.hopendialogue.net/

## Supplementary material

SUPPLEMENTARY FIGURE 1 Historical development of OD-services in different countries from 1990–2022.

SUPPLEMENTARY FIGURE 2 Dendrogram of the cluster analysis using Ward linkage.

SUPPLEMENTARY DATASHEET 1 HOPEnDialogue-Survey Questionnaire 2020.
