# Supplementary figures and images for: Open Dialogue services around the world: a scoping survey exploring organizational characteristics in the implementation of the Open Dialogue approach in mental health services
Source: Front Psychol. 2023 Nov 10;14:1241936. doi: 10.3389/fpsyg.2023.1241936 (PMC10668593; doi:10.3389/fpsyg.2023.1241936)

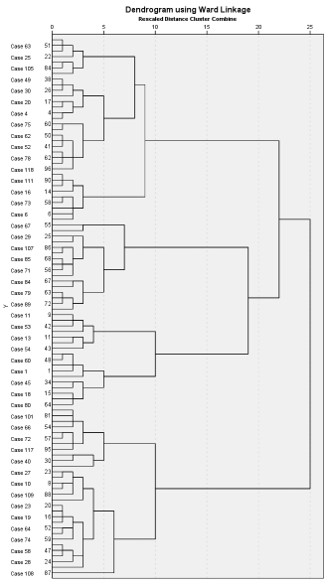

Supplement: SUPPLEMENTARY FIGURE 1 — Historical development of OD-services in different countries from 1990–2022. [file Image_1.jpg]

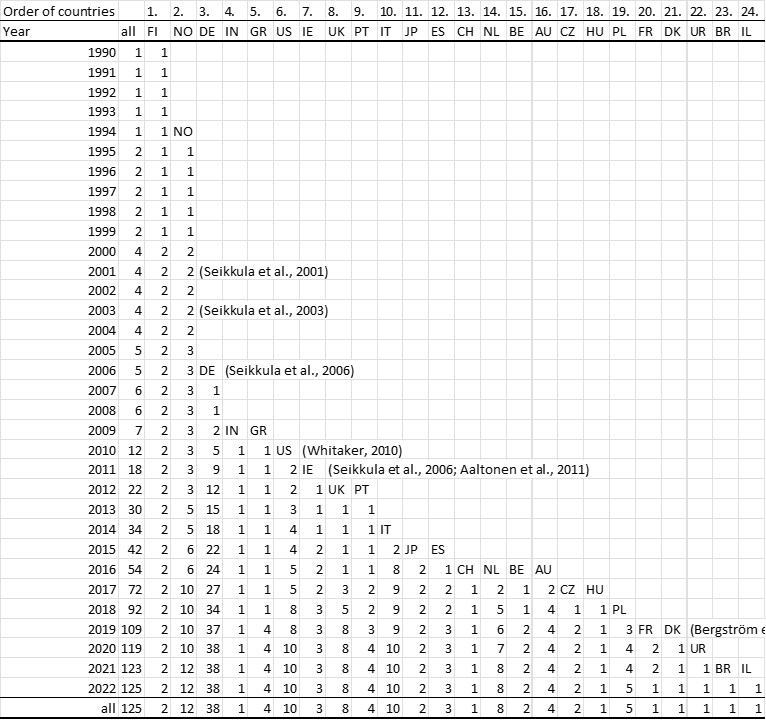

Supplement: SUPPLEMENTARY FIGURE 2 — Dendrogram of the cluster analysis using Ward linkage. [file Image_2.jpeg]
