# Supplementary material for: Open Dialogue services around the world: a scoping survey exploring organizational characteristics in the implementation of the Open Dialogue approach in mental health services
Source: Front Psychol. 2023 Nov 10;14:1241936. doi: 10.3389/fpsyg.2023.1241936 (PMC10668593; doi:10.3389/fpsyg.2023.1241936)
Supplement: SUPPLEMENTARY DATASHEET 1 — HOPEnDialogue-Survey Questionnaire 2020. [file Data_Sheet_1.PDF]

HOPENDIALOGUE

International HOPEnDialogue Survey

Thank you for your interest in taking the HOPEnDialogue Survey

This is an international survey for all services developing/practicing Open Dialogue. Please read the following information before taking the survey.

What data do we collect?

We collect information about your mental health service and the way you are practicing Open Dialogue.

We also collect your personal information to contact you in case there is a need to clarify something or to receive further details.

How do we collect your data?

You directly provide us all the data we collect, voluntarily completing this survey. Information is collected using Survey Monkey, which is compliant with the GDPR and adopts the following privacy policy: Survey Monkey privacy policy.

Taking part in the study involves responding to an online survey that takes approximately 45 minutes to complete. It will be possible to complete the survey between January and April 2020.

How will we use your data?

The results of the survey will assist us to determine the extent of Open Dialogue service development internationally and will inform the selection of the OD services which will be involved in the HOPEnDialogue study.

The results of the survey will be presented in an aggregated and anonymous form for open access scientific publications, presentations in scientific conferences and websites interested in the Open Dialogue approach.

How do we store your data?

The survey database will be deposited in the ISTC-CNR data repository and on a protected computer for the time needed for the HOPEnDialogue study, which we estimate will be concluded in six years.

How do we protect your data?

Information will be identified using a code (pseudo-anonymized). Only researchers of the HOPEnDialogue team will have access to your data. They adopt the "Guidelines for Research Integrity" produced by the CNR, approved on the 10 June 2015 and updated in 2019: https://www.cnr.it/en/ethics. The CNR registers all the activities related to data treatment (CNR's Circular n. 10/2018).

Who is responsible for data protection and treatment?

The Data controller is the Italian National Research Council (CNR), Piazzale Aldo Moro, 7, Rome. The Data Protection officer is Dr. Giuliano Salberini (CNR's Circular n. 27/ 2019).

The director of the ISTC-CNR, dr. Aldo Gangemi is responsible for data treatment (CNR's Circular n. 41/2018).

Dr. Raffaella Pocobello is the person authorized to the gathering and use of the data of this survey.

How to contact us?
Please contact dr Pocobello for any question, concern or complaint about your participation in this online survey. Email: raffaella.pocobello@istc.cnr.it ; Skype: raffaella.pocobello.

What are your data protection rights?
Your participation in this study is voluntary. You have the right:
- to ask further information;
- to refuse to answer questions and withdraw from the study at any time without having to give a reason;
- to access the information you provide for this survey, requesting a copy of your personal data;
- to correct any information you believe is inaccurate and ask us to include this into the survey;
- to request that we erase your personal data or restrict its processing;
- to lodge a complaint with the authorities.

How to be informed about the results of the survey?
The results of the survey will be presented on the HOPEnDialogue website, where materials and publications will be posted.

* 1. Clicking on the "agree" button below indicates that:
- you have read the above information
- you voluntarily agree to participate
- you are at least 18 years of age

◯ I agree

HOPENDIALOGUE

International HOPEnDialogue Survey

Contact information

* 2. You may wish to share general contact details for your organization or include the name of a specific contact person.

Name	
Surname	
Name of the service	
Service address	
City	
Postal Code	
Country	
Email address	
Telephone number	

International HOPEnDialogue Survey

General informations about the mental health service

* 3. In which sector is your service?

- Private
- Public
- Other (please specify)

* 4. Is it an inpatient or outpatient service, or both?

- Inpatient
- Outpatient
- Both
- Other (please specify)

* 5. Is your service a stand-alone service or integrated with other services?

- stand-alone service
- integrated service
- Other (please specify)

International HOPEnDialogue Survey

Information about your Open Dialogue service

From here on we will ask information about the part of your service that works with Open Dialogue

6. What age range of clients do your OD service work with?

- [ ] Under 18
- [ ] 18-24
- [ ] 25-34
- [ ] 35-44
- [ ] 45-54
- [ ] 55-64
- [ ] 65+

7. Which client groups does your OD service work with?

- [ ] Psychotic disorders
- [ ] Mood disorders
- [ ] Anxiety- and Fear-Related Disorders
- [ ] Disorders specifically assocciatred with stress
- [ ] Other disorders

8. How long has your OD service been established (years)?

____

9. If it is a not yet established OD service, please indicate the date when you anticipate starting

____

10. Please indicate the days (standard day) on which your service is open:

open

Time | AM/PM
hh mm | -

close

Time | AM/PM
hh mm | -

Please describe the criteria for entry to your Open Dialogue service and the treatment pathways:

11. If appropriate for your service, please indicate the dimension of the catchment area (number of persons served)

12. Sources of referrals

13. Points of acceptance

14. Progress and transition through the service

15. Point of discharge

16. Follow up

17. Please describe the aims and the therapeutic model/s of the work offered in your service (for example: does your service adopt primarily Open Dialogue or does integrate it with other models?)

International HOPEnDialogue Survey

Fidelity

Please consider now the clinical practice in your service, taking into account the overall functioning of your service in the past three months. Please indicate on a scale from "never" to "almost always" the extent to which the following statements capture the practice in your service.

* 18. The first meeting takes place within 24 hours after the request for help.

◯ Never ◯ Rarely ◯ Sometimes ◯ Frequently ◯ Almost always

* 19. The first meeting takes place within one week of the request for help.

◯ Never ◯ Rarely ◯ Sometimes ◯ Frequently ◯ Almost always

* 20. Network meetings are organized for all clients using the service.

◯ Never ◯ Rarely ◯ Sometimes ◯ Frequently ◯ Almost always

* 21. The service engages effectively for the whole treatment with clients and their support systems.

◯ Never ◯ Rarely ◯ Sometimes ◯ Frequently ◯ Almost always

* 22. The service actively seeks to involve family and care-givers in network meetings.

◯ Never ◯ Rarely ◯ Sometimes ◯ Frequently ◯ Almost always

* 23. Needs and plans of clients and their families are considered and shared during the meetings.

◯ Never ◯ Rarely ◯ Sometimes ◯ Frequently ◯ Almost always

* 24. Service adapts the intervention to the clients' needs.

◯ Never ◯ Rarely ◯ Sometimes ◯ Frequently ◯ Almost always

* 25. Service offers access to as many interventions necessary to meet the clients' needs (psychological, social, pharmacological, psychoeducational, peer-support interventions, etc.).

◯ Never ◯ Rarely ◯ Sometimes ◯ Frequently ◯ Almost always

* 26. Network meetings are established based on clients' wishes and take place at the convenience of the clients (home, community, mental health services, hospital, etc.).

◯ Never ◯ Rarely ◯ Sometimes ◯ Frequently ◯ Almost always

* 27. Staff members form a team for the entire treatment process.

◯ Never ◯ Rarely ◯ Sometimes ◯ Frequently ◯ Almost always

* 28. When a service user is re-referred to the service, the service makes active efforts to re-assign the professionals previous engaged in the service user's care.

◯ Never ◯ Rarely ◯ Sometimes ◯ Frequently ◯ Almost always

* 29. The professional responding to the first request for help is responsible for organizing the first network meeting.

◯ Never ◯ Rarely ◯ Sometimes ◯ Frequently ◯ Almost always

* 30. All service-users have at least a named staff member coordinating his or her care throughout their treatment.

◯ Never ◯ Rarely ◯ Sometimes ◯ Frequently ◯ Almost always

* 31. Discussions about the clients and their networks occur in their presence.

◯ Never ◯ Rarely ◯ Sometimes ◯ Frequently ◯ Almost always

* 32. Decisions and treatment plans, including the use of medications, are defined openly during the network meeting.

◯ Never ◯ Rarely ◯ Sometimes ◯ Frequently ◯ Almost always

* 33. There is an active effort of the team to avoid premature decisions or treatment plans.

◯ Never ◯ Rarely ◯ Sometimes ◯ Frequently ◯ Almost always

* 34. Professionals share their own personal experiences (self-disclosure) in network meetings and supervisions if deemed relevant and appropriate.

◯ Never ◯ Rarely ◯ Sometimes ◯ Frequently ◯ Almost always

International HOPEnDialogue Survey

Staffing and service capacity

35. Please provide your best estimate of the current numbers of staff members included in your OD service or team (for each role):

Nurses

Occupational Therapists

Peer-support workers

Psychiatrists

Psychologists/ Psychotherapists

Social Workers

Support Workers

Others

36. On average how many hours a week do staff members usually engage in OD practice?

37. Please estimate:

team caseload currently (the number of persons/families who engage in your OD service)

the maximum caseload for your service

Routine data collection & Research

* 38. Please indicate if your routine data collection system includes (you can select more than one option):

- [ ] Gender
- [ ] Age
- [ ] Information on mental health, including symptoms and diagnosis
- [ ] Psychiatric history
- [ ] Medications use & prescriptions
- [ ] Alcohol and drug use
- [ ] Information on social circumstance (this might include info on living situation, employment, family contact, financial issues, relationship status, etc)
- [ ] Routine Outcomes measurement (e.g. clinical or functioning scales, relapse indicators)
- [ ] Other (please specify)

39. Does your service have any kind of formal feedback process to evaluate the clients' and carers' experience or satisfaction?

○ Yes ○ No

40. Is the data collected reported and used for service evaluation?

○ Yes

○ No

41. Please indicate if your Open Dialogue service is involved in

- [ ] Audits
- [ ] Evaluations
- [ ] Quality Improvement programmes
- [ ] Research programmes
- [ ] Other (please specify)

42. Does your service include an R&D (Research and Development) Unit?

- ○ Yes
- ○ No

43. Does your service collaborate with Universities/Research institutions?

- ○ Yes
- ○ No

If yes, please indicate

44. Has your service been involved in research programs?

- ○ Yes
- ○ No

45. If yes, please include a list of the research programs

46. Should there be articles or other materials developed by/related to your Open Dialogue service, please include a list here

Open Dialogue Training

47. Please indicate the number of staff trained in your service and the type of training(s) that they have undertaken

Trainers' Training Programme

Full 3/4 Year Open Dialogue Practitioner Training

Open Dialogue Practitioner Foundation Training

International Certification Training in Dialogic Practice

Peer-Supported Open Dialogue, Social Network and Relationship Skills Foundation Training

* 48. Please indicate the number of staff trained and whether trainings are in progress or have been completed):

Number of staff trained

Number of staff-Training in progress

49. Did the training include some self-work on participants' family of origin?

- ◯ Yes, for all the practitioners trained
- ◯ Yes, for the majority of practitioners trained
- ◯ Yes, but only for few practitioners
- ◯ No

* 50. Please indicate which of these options is valid for your Open Dialogue service

- ◯ All clinical staff have completed or are undergoing a recognized Open Dialogue training program.
- ◯ All clinical staff, with a small number of exceptions (e.g. a couple of members of staff who have recently joined, but are expecting to start training soon), have completed or are undergoing a recognized Open Dialogue training.
- ◯ The majority of clinical staff have completed or are undergoing a recognized Open Dialogue training, and most of the remaining staff are due to be trained soon.
- ◯ Less than half of clinical staff have completed or are undergoing a recognized Open Dialogue training.

Open Dialogue Supervision

* 51. Is supervision in place to help clinicians reflect on/develop their Open Dialogue practice?

◯ Yes ◯ No

52. Does your supervision include:

- ☐ brief mindfulness practice;
- ☐ practitioner reflections (team members share personal reflections in pairs/groups on the unique challenges they are experiencing in their interaction with the families/networks they are seeing);
- ☐ these reflections are observed and then reflected on by other team members;
- ☐ final reflections (original pair/group share a final reflection at the end).

* 53. Supervision takes place:

- At least weekly
- At least once a month
- At least once every three months

* 54. Team meetings to reflect on Open Dialogue practice take place:

- At least weekly
- At least once a month
- At least once every three months

Experts by experience/peers involvement

* 55. Do experts by experience contribute to your Open Dialogue service?

- Yes
- No- Please specify why (e.g., no budget, no interest, no trained peers available, legal issues, etc.)

56. Please indicate in which of the following activities experts by experience are involved

- Development & planning
- Training (as trainees)
- Evaluation & assessment
- Training (as trainers)
- Delivery of care
- Other (please specify)

57. How many experts by experience are practicing Open Dialogue?

58. Do experts by experience take part in all network meetings?

◯ Yes ◯ No

59. Is the expert by experience/peer role in the team formally recognized?

◯ Yes ◯ No

60. Are experts by expirience volunteers?

◯ Yes ◯ No

61. Are experts by experience/peers paid workers?

◯ Yes ◯ No

62. What is the role of experts by experience in network meetings?

- [ ] Facilitators/moderators
- [ ] Engaging in reflections
- [ ] Joining as support for the service user or family network
- [ ] Other (please specify)

63. Are experts by experience involved in supervision as the other members of the team?

◯ Yes ◯ No

64. Do experts by experience receive psychological support or dedicated supervision?

◯ Yes ◯ No

65. Do you have any initial thoughts to share about the possible participation of your service in the next steps of the HOPEnDialogue study?

HOPENDIALOGUE

International HOPEnDialogue Survey

Acknowledgment

This questionnaire was adapted from the COM-FIT Questionnaire developed for the ODDESSI PROGRAMME by Alvarez-Monjaras, Lotomore, Clarke, Razzaque & Pilling (2019). The lists of Open Dialogue training have been quoted from Open Dialogue Uk.

Thank you for your collaboration!
We wish to meet you on June 21st and 22nd 2021 in Rome for the International Open Dialogue Meeting!

< CLICK ON FINISH TO SEND YOUR SURVEY >
